# Characterization of a novel disease-causing mutation in exon 1 of SH2D1A gene through amplicon sequencing: a case report on HLH

**DOI:** 10.1186/s12881-017-0376-9

**Published:** 2017-02-14

**Authors:** Shiyuan Zhou, Hongyu Ma, Bo Gao, Guangming Fang, Yi Zeng, Qing Zhang, GaoFu Qi

**Affiliations:** 1 Henan Research Institute for Population and Family Planning, Zhengzhou, China; 2 Key Laboratory of Birthdefects Prevention, National Health and Family Planning Commission, #26 Jingwu Road, Zhengzhou, Henan China; 3 Thermo Fisher Scientific, Building 6, N0.27, Xin Jinqiao Rd, Pudong, Shanghai, China; 4 Department of Laboratory Medicine, Taihe Hospital, Hubei University of Medicine, Shiyan, China; 5 Department of Clinical Medicine, Zhengzhou University, No.100 Science Avenue, Zhengzhou, China; 6 Institute of System Biology, Jianghan University, Sanjiaohu Rd, Wuhan, Hubei China

**Keywords:** HLH, *SH2D1A*, Amplicon sequencing, Mutation, Genetic analysis

## Abstract

**Background:**

Hemophagocytic lymphohistocytosis (HLH) is a rare but fatal hyperinflammatory syndrome caused by uncontrolled proliferation of activated macrophages and T lymphocytes secreting high amounts of inflammatory cytokines. Genetic defect is a common cause of HLH. HLH is complicated to be diagnosed as there are many common symptoms with other disorders.

**Case presentation:**

Here we report on an HLH case caused by 1 bp deletion in gene *SH2D1A*. Patient was a 3-years-old boy and had fever for more than 8 days. Splenomegaly and hemophagocytosis in bone marrow were observed in examination. The results of the blood analysis suggested the diagnosis of HLH. Genetic test based on high throughput amplicon sequencing was then conducted by targeting all six known HLH-causing genes simultaneously. It took only one single day to accomplish the amplicon sequencing library preparation, sequencing and data analysis. Finally, a novel 1 bp deletion in gene *SH2D1A* was discovered. The result was also confirmed by Sanger sequencing. The result of the genetic test served as a good basis for further diagnosis of HLH.

**Conclusion:**

This is the first case that the disease-causing genetic defect of HLH was quickly determined by high throughput amplicon sequencing. This diagnosis was also confirmed by Sanger sequencing and cross-validated by blood analysis and other clinical criteria. This case suggests that genetic test based on amplicon sequencing is a powerful tool for diagnosis of HLH and other diseases caused by genetic defect.

**Electronic supplementary material:**

The online version of this article (doi:10.1186/s12881-017-0376-9) contains supplementary material, which is available to authorized users.

## Background

Hemophagocytic lymphohistocytosis (HLH) is a potentially fatal disease with severe systemic syndrome, which is caused by uncontrolled proliferation of activated macrophages and T lymphocytes, and impaired regulation of the immune system. The first case was reported at 1952 by Farquhar et al., and the mortality rate of HLH ranged from 22 to 60% [1, 2]. Many reports have revealed that HLH could affect all age groups, from preterm neonates to elderly adults [3]. The overall occurrence rate might be underestimated as it could be diagnosed as other disease sharing similar symptom [3, 4].

Usually HLH is rapidly fatal unless treated aggressively [5]. Rapid definitive diagnosis and appropriate treatment are extremely important for life-saving and improved prognosis for patients. Established clinical and laboratory criteria help significantly in accurate clinical diagnosis, but most of them are time-consuming. Furthermore, they are not sensitive and specific enough for HLH diagnosis, so the results of these tests may be supportive of, but not diagnostic of, HLH. Therefore, a gene mutation analysis should be the gold standard for making a definitive diagnosis. To date, a total of 6 causative genes have been identified, including *SH2D1A*, *PRF1*, *UNC13D*, *STX11*, *STXBP2* and *XIAP*, and a few mutations in these genes have been reported to be the common cause of HLH [6–9]. Usually, these genes were checked one by one to detected mutations. Here, high throughput genetic analysis was conducted to detect mutations in all six genes simultaneously with amplicon sequencing in a HLH patient. We identified a novel mutation in gene *SH2D1A*, which was also confirmed by Sanger sequencing and this patient was diagnosed as HLH finally. This indicated that amplicon sequencing was a powerful tool in diagnosis of genetic disease, especially for cases involved multiple candidate causative genes.

## Case presentation

The study was approved by the institute ethics committee of Key Laboratory of Birthdefects Prevention, National Health and Family Planning Commission, Zhengzhou, China. Informed consent from the parents was obtained before collecting blood samples. A 3-years-old boy with HLH was diagnosed based on blood analysis and genetic detection. The causing mutation was a homozygous 1 bp deletion (c.92delT) in the first exon of SH2D1A and this deletion was validated by Sanger sequencing. This patient and his parents are all Han Chinese from Henan province of China.

### Clinical data

**Fig. 1:**
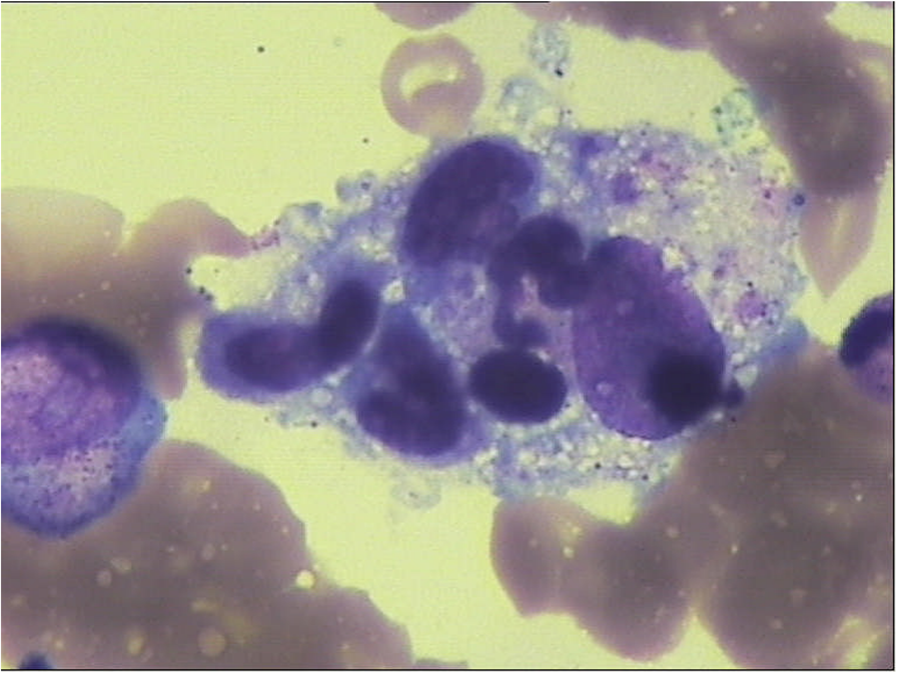
The bone marrow examination. Phagocytosis could be clearly observed in the boon marrow

The 3-years-old patient had fever for more than 8 days with unknown origin when he was admitted to Key Laboratory of Birthdefects Prevention, National Health and Family Planning Commission, Zhengzhou, China. He had taken penicillin for 5 days before admission. On examination, the temperature was measured to 39.5 °C. No other respiratory symptoms but tachypnea presented in physical examination. The central nervous system was normal. Ultrasound examination revealed splenomegaly. Blood analysis was conducted at Key Laboratory of Birthdefects Prevention and hemoglobin was measured to be 90 g/L. Platelets, Neutrophils and Fibrinogen were measured to be 22× 10^9^/L, 7.54× 10^9^/L and 3.42 g/L, respectively. Low NK-cell activity (5.05%) and low Plasma albumin (18.9 g/L) were also observed. The bone marrow examination suggested hemophagocytosis without any evidence of malignancy [Fig. 1]. The results of the blood analysis and clinical features suggested the diagnosis of HLH based on HLH-2004 guidelines [10]. A two-generation pedigree including the patient and his parents was generated.

### Detection of mutation of six genes

Genomic DNA was purified from peripheral blood mononuclear cells PBMC with QIAamp Blood Kit (Qiagen, Hilden, Germany) according to the protocol. Multiplexed primers were designed for 6 disease-causing genes (*SH2D1A*, *PRF1*, *UNC13D*, *STX11*, *STXBP2* and *XIAP*) with Ion AmpliSeq™ Designer (https://www.ampliseq.com) and a total of 64 exons were covered by these primers, including the whole coding regions of these genes and splicing sites for each exon-intron boundaries. Multiplexed polymerase chain reaction (PCR) amplification was conducted and the PCR product was ligated with adapters, purified and then sequenced by proton sequencing based S5XL genetic analyzer (Applied Biosystems®, Life Technologies, Grand Island, NY, USA) and mutations were analyzed using VariantCaller V1.0 software embedded in the Torrent Suited software (Life Technologies). Primer information could be found in supplementary file [see Additional file 1].

The average base coverage depth for the whole target regions was 1,515 and more than 97.13% of all sites had coverage of more than 100-fold, which indicated a good performance of multiplexed PCR. A total of 19 SNPs (17 heterozygous SNPs and two homozygous SNPs) were detected in all five genes except *XIAP* and no insertion was found in all genes [Table 1]. One homozygous deletion was identified in the first exon of *SH2D1A*. Among all 19 SNPs, 11 were known mutations recorded in the NCBI refSNP database and no known cases have been reported to be associated with these SNPs. For the remaining eight novel SNPs, no damage-causing effect was found by searching literatures and databases, as well as domain analysis for each protein, including six synonymous mutations and two missense mutations (c.2599A > G in the 27th exon of gene UNC13D, c.1576A > G in the 18th exon of gene STXBP2). In summary, no novel and known SNPs had been proved to be disease-related mutations.

**Table 1:** All variation sites detected by amplicon sequencing

gene-exon gene-exon	position	Type	Zygosity	Reference	Variant	Freqence of variant	coverage^a^	ID in refSNP^b^	comment
STX11-exon2	c.*70G > A	non-coding region	Homozygous	G	A	100	740	rs3734228	none disease-causing
PRF1-Exon3	c.900C > T p.His300His	synonymous mutation	Homozygous	G	A	100	420	novel	none disease-causing
PRF1-Exon3	c.822C > T p.Ala274Ala	synonymous mutation	Heterozygous	G	A	51.1	999	novel	none disease-causing
UNC13D-exon32	c.3198A > G p.Glu1066Glu	synonymous mutation	Heterozygous	T	C	51.1	963	novel	none disease-causing
UNC13D-exon29	c.2710-48C > T	intron	Heterozygous	G	A	49.2	1000	rs2290768	none disease-causing
UNC13D-exon27	c.2599A > G p.Lys867Glu	missense	Heterozygous	T	C	48.3	986	novel	none disease-causing
UNC13D-exon24	c.2299-46C > T	intron	Heterozygous	G	A	44.5	999	rs7212635	none disease-causing
UNC13D-exon21	c.1977C > T p.Thr659Thr	synonymous mutation	Heterozygous	G	A	38.1	1000	novel	none disease-causing
UNC13D-exon20	c.1728-48 T > C	intron	Heterozygous	A	G	47	985	rs3744024	none disease-causing
UNC13D-exon18	c.1596 + 36A > G	intron	Heterozygous	T	C	45.8	998	rs3744026	none disease-causing
UNC13D-exon11	c.888G > C p.Pro296Pro	synonymous mutation	Heterozygous	C	G	51.8	998	novel	none disease-causing
UNC13D-exon7	c.570-60 T > G	intron	Heterozygous	A	C	52.7	995	rs8067076	none disease-causing
UNC13D-exon1	c.117 + 30G > A	intron	Heterozygous	C	T	48.2	842	rs3744011	none disease-causing
STXBP2-exon2	c.38-7C > T	intron	Heterozygous	C	T	59.5	79	rs8104339	none disease-causing
STXBP2-exon15	c.1356 + 18A > G	intron	Heterozygous	A	G	47.2	998	rs889187	none disease-causing
STXBP2-exon15	c.1356 + 77A > G	intron	Heterozygous	A	G	46.9	997	rs710951	none disease-causing
STXBP2-exon16	c.1443 T > C p.Asp481Asp	synonymous mutation	Heterozygous	T	C	52.9	357	novel	none disease-causing
STXBP2-exon18	c.1576A > G p.Ile526Val	missense	Heterozygous	A	G	46.5	185	novel	none disease-causing
STXBP2-exon18	c.1696 + 20A > G	intron	Heterozygous	A	G	51.7	201	novel	none disease-causing
SH2D1A-exon1	c.92delT p.Leu31Argfs*50p.	frameshift mutation	Homozygous	T	-	100	864	novel	disease-causing

^a^ Coverage means the overall depth of this site by amplicon sequencing reads

^b^ If a known variant was detected for the targeted gene,its accession No kept in NCBI was listed, or we marked it as novel

**Fig. 2:**
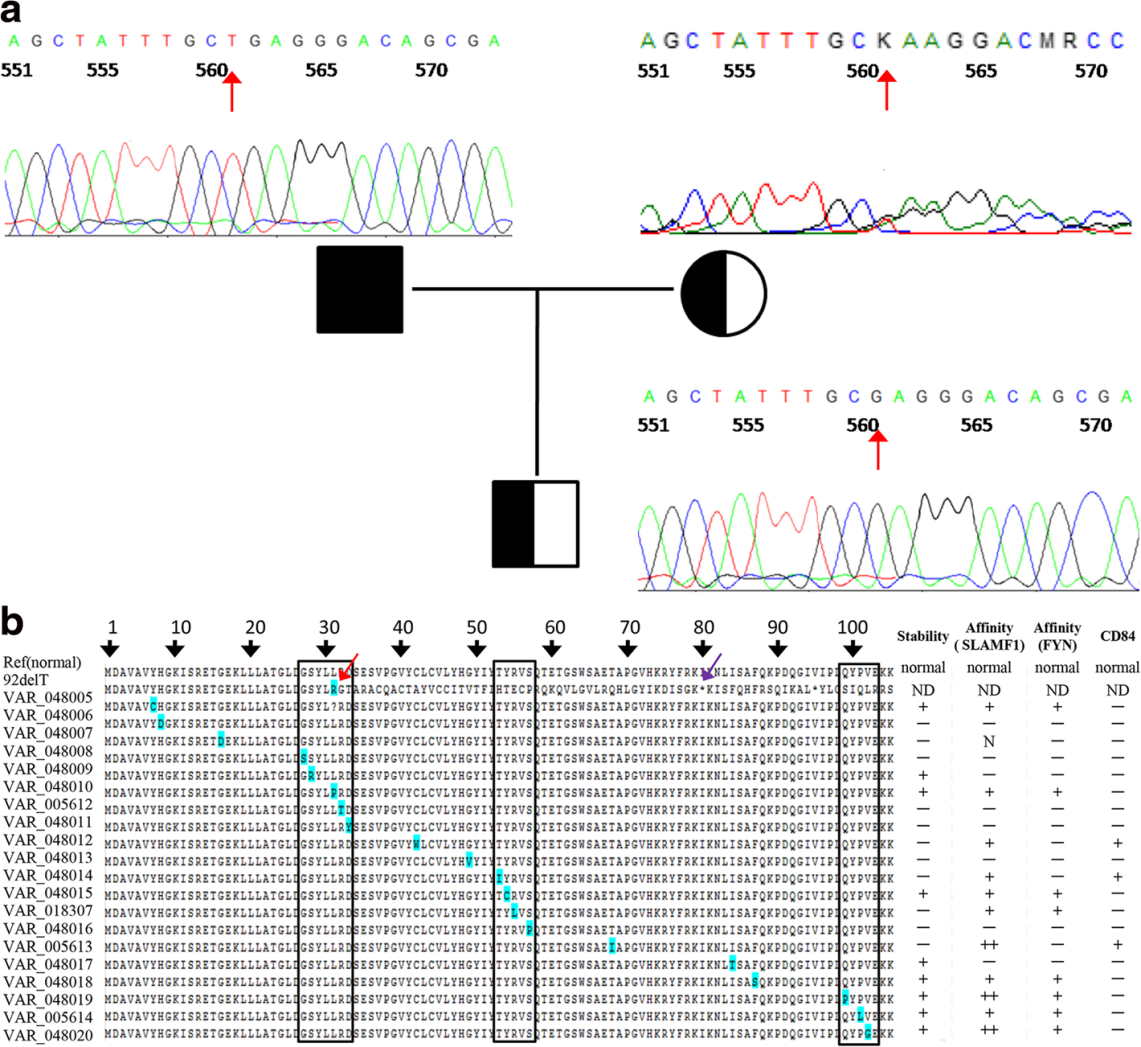
Mutations in the gene *SH2D1A*. **a** Sanger sequencing results of the 3-years-old child (*underside*), his father (*top left*) and his mother (*top right*). Mutation position is marked with *red arrow*. The results show heterozygosity for this mutation in his mother, but wild type sequences in his father. As indicated, deleted nucleotide caused a frameshift in the corresponding sequence. b) Mutations recorded in SWISSPROT. All mutations are marked in *blue* and each mutational hotspot is indicated by *black box*. Almost all the mutations located in these three regions have been reported to be disease-causing defects. The novel deletion detected in this study is marked with *red arrow* and the premature stop codon is marked with *purple* arrow. “VAR_048***” is the accession No in SWISSPROT, “ND” represents for “Not detected”, “+” indicates that the mutation could reduce protein stability or interaction with other protein, “++” indicates a significant reduction and “N” indicates totally abolished interaction with SLAMF1. “-“means no data available

A novel homozygous 1 bp deletion (c.92delT) was identified in exon 1 of *SH2D1A*, which was located on the X chromosome (at Xq25) [11]. This deletion was confirmed by Sanger sequencing and also detected in the patient’s mother, but not in his father [Fig. 2a]. This indicated that it was not a *de novo* variation, but inherited from the mother. Interestingly, this variation in the mother was found to be heterozygous based on Sanger sequencing [Fig. 2a]. The deletion resulted in an introduction of a premature stop codon and produced a truncated protein (79aa, p.Leu31Argfs*50) [Fig. 2b]. SH2D1A is a signaling lymphocytic activation molecule (SLAM)-associated protein (SAP), and it is one of the known disease-causing genes associated with immunodeficiency, which led to X-linked lymphoproliferative disease (XLP) [12]. The affect of the mutations occurred in *SH2D1A* typically manifested in childhood or early adolescence and it was estimated that it affected 1–3 per million boys [13]. A lot of mutations in *SH2D1A* have been identified and more than 200 cases related with these mutations has been reported [14, 15]. We found three mutational hotspots in the *SH2D1A* gene according to the records in SWISSPROT. The novel 1 bp deletion (c.92delT) presented here was located in the first one of these mutational hotspots.

## Discussion

In this case, two other possible diseases have been noted considering that *SH2D1A* mutation could involve in rheumatoid arthritis (RA) [16] and systemic lupus erythematosus [17], but both of these diseases were excluded. Firstly, splenomegaly and Hemophagocytosis in bone marrow were observed in examination, and all blood analysis showed that some key indexes fitted in with the diagnosis of HLH based on HLH-2004 guidelines, which has been revised at 2007. If the child had RA, the temperature will not be as high as 39.5 °C, but just low-grade fever. And it should not be systemic lupus erythematosus, because no obvious symptoms could be found in physical examination. The central nervous system was also normal. Together with the identification of 1 bp deletion in exon of *SH2D1A*, a diagnosis of HLH could be accepted. However, it is interesting to note that the gene *SH2D1A* is located on the X chromosome [11] and Sanger sequencing revealed that the mutation was heterozygous in the mother, which might explain why his mother had no obvious symptoms of HLH. This also indicated that the case presented here was X-linked genetic disease, especially considering the incidence of the patient. As soon as sequencing result was acquired and HLH diagnosis was confirmed, the patient received full course of dexamethasone combining with VP-16 chemotherapy, according to the guideline of HLH-2004 protocol [10]. The clinical manifestation quickly stabilized and currently he is under treatment for further HSCT procedure.

*SH2D1A* encodes the signaling lymphocytic activation molecule (SLAM)-associated protein (SAP), which interacts with members of the CD2 subset of the Ig superfamily of cell-surface receptors [12]. It has been reported that there are three putative acting mechanisms that could cause XLP due to mutation in *SH2D1A*, including reduced gross expression, reduced ability binding to SLAM family receptors and inability to activate signal transduction downstream of the SLAM receptor–SAP complex efficiently [18]. In the present case, it is believed that no functional protein was generated due to deletion of 92th base (T), which could lead to functional defects in natural killer (NK) cells. NK cells are required for clearance of viral infection as well as regulation and termination of the inflammatory response [19]. Phagocytes, neutrophils and NK cells constitute the innate immune system and persistent antigen exposure will keep them active. These activated cells released proimflammatory cytokines such as TNF-α, IL-1 and IL-6, which could cause fever, hyperferritinemia and other symptoms.

HLH occurs predominantly in infants and very young children. Based on the mechanism of morbidity, HLH can either be genetic, or acquired due to viral infections, malignancies or rheumatic diseases. According to classification of histiocytic disorders, HLH is subdivided into familial HLH (FHLH) and secondary HLH (sHLH) [2]. Based on genetic etiology, FHLH has been subcategorized into 5 subtypes, i.e., FHLH-1 to FHLH-5 [20]. However, these disorders may be difficult to be distinguished from each other clinically. For example, the clinical symptoms of familial HLH usually become evident within the first 2 months of life, but most clinicians cannot distinguish FHL from other disorders if the patient is the first child or in the absence of affected siblings.

A set of clinical and laboratory criteria have been established for accurate diagnose of HLH [10], such as prolonged high-grade fever, progressive cytopenias, hepatosplenomegaly with liver dysfunction, skin rash, coagulopathy and variable neurologic symptoms, and these criteria have been widely used in clinical practice. The pathologic findings of activated macrophages, engulfing erythrocytes, leukocytes, platelets and their precursor cells were also important for diagnosis of HLH. However, not all HLH cases present these clinical manifestations in the initial stage [21], and the organs involved may be different [22]. Especially, it is has been reported that HLH may also commonly occurred in a setting of rheumatologic illness [23].

In practice, analysis of bone marrow aspirate is commonly used for HLH diagnosis, which only has a sensitivity of 60% [24]. Therefore, a negative bone marrow analysis result should not completely rule out HLH. A set of other rapid diagnostic tests have been proposed to aid the diagnosis of HLH, including Ferritin, soluble IL-2 receptor, IFN-g, IL-10 and other proinflammatory cytokines [10, 25, 26]. All these indices have been reported to be useful in diagnosing HLH. Novel methods have also been developed for the early and rapid detection of patients. For example, flow cytometry assay has been used to evaluate the expression of lymphoid SAP (XLP1), perforin and XIAP (XLP2) [27], and it could also detect revertants and somatic mutations [28]. It has to be noted that all these criteria and methods are proposed based on physiological indices, not genetic, and they have limited sensitivity and specificity for HLH. On the other side, screening of mutations in all these 6 causative genes are generally conducted by Sanger sequencing, and constrained by its low throughput. Now high throughput sequencing is cost-effective and time-effective for genetic testing, and it could detect all target loci simultaneously in 1 day, even most proportion of whole genome if necessary. Together with highly multiplexed PCR technology, it could identify all kinds of variants with high accurate by covered the target region (i.e., causative genes) with a proper depth. This may help to reduce the time consumed by differential diagnosis and make the definitive diagnosis rapidly. This is extremely important for life-saving and getting improved prognosis by giving patients appropriate treatment as soon as possible. In order to prevent misdiagnosis and give as much information as possible for clinicians, it will be helpful and necessary to do this genetic analysis immediately with amplicon sequencing after child is born.

## Conclusion

In summary, 6 HLH-related genes were tested at once in a suspected HLH patient and a novel homozygous 1 bp deletion (c.92delT) in exon 1 of *SH2D1A* was indentified. The diagnosis of HLH was further confirmed by this result. Most HLH occurred in childhood and the survival rate is very poor, even with treatment [29]. Rapid definitive diagnosis and appropriate treatment are extremely important for life-saving and prognosis-improving. This indicated that amplicon sequencing is necessary for rapid and accurate screening of mutations in the diagnosis of genetic disease, especially those clinically indistinguishable disorders. Other methods based on DNA and proteins analysis have been reported for the detection of *SH2D1A* mutation [28, 30], but none could be used to detect all possible genes simultaneously and rapidly. Ultimately, this case proposed a novel method to identify HLH-related mutations, and improve the understanding of their roles during the regulation of host antiviral immune responses. It will also facilitate development of novel therapies for these rare but devastating disorders.

## Additional file

Additional file 1
**Table S1**: List of primers that were used for amplicon sequencing. (XLSX 19 kb)

## Abbreviations

FHLH: Familial hemophagocytic lymphohistocytosis
HLH: Hemophagocytic lymphohistocytosis
sHLH: Secondary hemophagocytic lymphohistocytosis
XLP: X-linked lymphoproliferative disease
